# The impact of mental disorders on the contemporary world of work and the challenges of working as a health promoter

**DOI:** 10.47626/1679-4435-2024-222

**Published:** 2024-09-24

**Authors:** Sergio Roberto de Lucca

**Affiliations:** Department of Collective Health, School of Medical Sciences, Unicamp

Dear readers of the Brazilian Journal of Occupational Medicine (Revista Brasileira de Medicina do Trabalho, RBMT), I had the privilege of being invited to the editorial of this issue number 2 of volume 22 of our Journal, which contains relevant articles on the impacts on the physical and mental health of outsourced workers, health care professionals and workers, bank employees, beauty salon staff, transport workers, and truck drivers, as well as presenteeism that affects teachers and employees.

Since the 1990s, there has been a steep rise in mental and behavioral disorders (MBDs) and work-related mental disorders (WRMDs) in Brazil and worldwide, aggravated during and after the COVID-19 pandemic. Data from the World Health Organization (WHO) estimate that 15% of working-age adults have a mental disorder. Globally, 280 million people live with depression, 301 million people live with anxiety, 64 million people live with a serious mental illness, and 703,000 people die by suicide annually.^1^

The occurrence of MBDs and WRMDs inflicts damage to the mental health of the workforce and accompanies changes in the world of work, being aggravated with the advent of globalization and new technologies. Work intensification, practices of psychological violence to achieve goals, individual performance appraisal – which encourages individualism and competition between workers – and other work-related psychosocial factors,^2^ such as intrusion of work into private life, are other relevant aspects of contemporary work.

This issue of RBMT highlights the importance of psychosocial factors at work and outside work in the genesis of these events, which can trigger occupational stress and psychological distress, burnout syndrome, depression, and post-traumatic stress disorder (PTSD),^3^ in addition to the association with musculoskeletal disorders and the occurrence of presenteeism and increased absenteeism from work.

Workers’ decreased well-being and quality of life resulting from mental disorders leads to temporary or permanent work disability and productivity loss. Furthermore, a part of these events triggered or aggravated by work are preventable. In this context of precarious work and social relations in public and private organizations, it is expected that occupational physicians and other health professionals are able to diagnose and report these events, implementing individual and collective promotion and prevention actions toward a healthier work environment.

Faced with this competitive scenario and the precariousness of work and loss of solidarity in work relationships, in which everyone is suffering, most organizations, including human resources, occupational physicians, and health professionals, have difficulty managing adequately to preserve the mental health of workers under their responsibility.

In this context, the guidelines of the International Labor Organization (ILO)/WHO^1,4^ provide recommendations for “work-related mental health promotion and prevention,” pointing to a model of management and responsibilities of all actors involved (companies, leaders, employees, team of occupational physicians, and other professionals) to promote a healthy and more productive environment, with emphasis on organizational actions and interventions and training for leaders and other workers. This model presupposes a tripod of primary actions (identifying and acting on psychosocial risk factors), secondary actions (reducing emotional distress and providing support to those who are ill), and tertiary actions (those who return to work) that must reach all levels of the organization.

Although work may be a source of either pleasure or dissatisfaction, distress, and illness, what differentiates a transformative work from a distressful work is the perspective of thinking of workplaces as health promoters rather than disease producers. Creative actions at work enable changes in distress, contributing to a positive structure of identity and an increase in workers’ resilience in dealing with various forms of psychological and bodily imbalances.^5^

For this to occur and work to promote health, it is necessary to change the culture of contentment, which values commitment, infectious enthusiasm, and employees’ perfect health. Instead, or despite this, our biggest challenge is to ensure that workplaces can reestablish the inherent moral values of human beings, freedom, sociability, and awareness of self and others, based on respect, recognition, and relationships of mutual trust.

There is still hope that we can build workplaces where having autonomy to act to do a good job is possible, the workload (physical and emotional) is appropriate for the worker’s individual capacity, and people’s dignity is respected.

Enjoy your reading!
